# Study on the risk of coronary heart disease in middle-aged and young people based on machine learning methods: a retrospective cohort study

**DOI:** 10.7717/peerj.14078

**Published:** 2022-11-09

**Authors:** Jiaoyu Cao, Lixiang Zhang, Likun Ma, Xiaojuan Zhou, Beibei Yang, Wenjing Wang

**Affiliations:** Department of Cardiology, The First Affiliated Hospital of USTC, Division of Life Sciences and Medicine, University of Science and Technology of China, Hefei, Anhui, China

**Keywords:** Coronary heart disease, Young and middle-aged people, Logistic regression analysis, BP neural network, Random forest, XGBoost

## Abstract

**Objective:**

To identify coronary heart disease risk factors in young and middle-aged persons and develop a tailored risk prediction model.

**Methods:**

A retrospective cohort study was used in this research. From January 2017 to January 2020, 553 patients in the Department of Cardiology at a tertiary hospital in Anhui Province were chosen as research subjects. The research subjects were separated into two groups based on the results of coronary angiography performed during hospitalization (*n* = 201) and non-coronary heart disease (*n* = 352). R software (R 3.6.1) was used to analyze the clinical data of the two groups. A logistic regression prediction model and three machine learning models, including BP neural network, Extreme gradient boosting (XGBoost), and random forest, were built, and the best prediction model was chosen based on the relevant parameters of the different machine learning models.

**Results:**

Univariate analysis identified a total of 24 indexes with statistically significant differences between coronary heart disease and non-coronary heart disease groups, which were incorporated in the logistic regression model and three machine learning models. The AUCs of the test set in the logistic regression prediction model, BP neural network model, random forest model, and XGBoost model were 0.829, 0.795, 0.928, and 0.940, respectively, and the F1 scores were 0.634, 0.606, 0.846, and 0.887, indicating that the XGBoost model’s prediction value was the best.

**Conclusion:**

The XGBoost model, which is based on coronary heart disease risk factors in young and middle-aged people, has a high risk prediction efficiency for coronary heart disease in young and middle-aged people and can help clinical medical staff screen young and middle-aged people at high risk of coronary heart disease in clinical practice.

## Introduction

Coronary heart disease (CHD) is the world’s leading cause of death. Its incidence and fatality rates are higher in Asian countries than in Western countries (Vaisi-Raygani et al., 2010; Hata & Kiyohara, 2013). Most of the previous epidemiological data came from the elderly (>65 years old), but due to obesity and poor lifestyle, the incidence rate of CHD increased rapidly in young and middle-aged patients (Che et al., 2013). The Framingham Heart study reported the 10-year incidence rate of myocardial infarction (MI) in patients under 55 years old, 51.1/1,000 in men and 7.4/1,000 in women (Kannel & Abbott, 1984). (However, the literature on CHD and MI in young and middle-aged patients ≤65 years old is insufficient. The consequences of MI can be devastating, especially for young and middle-aged patients, because it has a greater potential impact on the patient’s psychology, work ability and socio-economic burden. Previous studies have pointed out the differences between young and elderly MI patients. Compared with elderly MI patients, young MI patients have a larger proportion of men, a higher incidence of smoking and hyperlipidemia, a lower incidence of CHD, diabetes and hypertension, and their prognosis is better than that of elderly patients (Afifi, 2006; Chouhan, Hajar & Pomposiello, 1993). Therefore, it is imperative to evaluate the risk factors of these CHD patients.

Considering that there are many middle-aged and young people with CHD in recent years (Yunjun, Yanan & Quan, 2017), and as the main labor force of society, middle-aged and young people are at the core of work and family. If accompanied with CHD, it will have a great impact on their work and life, increase the economic burden and bring calm pressure to the society (Cuilu, 2022). Therefore, it is of great significance to screen out the middle-aged and young people with high risk of CHD and take active and effective prevention and control measures. In recent years, many scholars have found that exploring new models of disease diagnosis based on machine learning algorithm has achieved good results in disease prediction and diagnosis (Dinh et al., 2019; Seo et al., 2019; Farran et al., 2019). Considering the harm of coronary heart disease in young and middle-aged people and the importance of early warning, this study used machine learning algorithm to establish an individual risk prediction model of coronary heart disease in young and middle-aged people, in order to provide an auxiliary diagnosis method for coronary heart disease in young and middle-aged people and reduce the risk of coronary heart disease in young and middle-aged people.

## Data and methods

### Data sources

This study is a retrospective cohort study, 553 patients in the Department of Cardiology of a tertiary hospital in Anhui Province from January 2017 to January 2020 were taken as the research object, including 201 middle-aged and young people with coronary heart disease as the coronary heart disease group and 352 people without coronary heart disease as the non-coronary heart disease group. Diagnostic criteria of coronary heart disease: (1) symptoms of angina pectoris or MI attack; (2) ECG showed myocardial ischemia changes; (3) The operation items include coronary angiography. The coronary angiography shows that there is stenosis in at least one main branch of the left main artery, left anterior descending artery, left circumflex artery or right coronary artery, and the stenosis is more than 50%, and the patient is diagnosed as coronary heart disease after discharge. The medical ethics committee of the First Affiliated Hospital of the University of Science and Technology of China gave their approval to this study (ID: 2022-RE-009). The subjects’ informed consent was not required because this was a retrospective study and the data was analyzed anonymously.

### Inclusion and exclusion criteria of the study population

Inclusion criteria: (1) The patient had no history of coronary heart disease; (2) Age 18-65 years old; (3) No mental illness. Exclusion criteria: (1) Combined with other acute and chronic infectious inflammation, cerebrovascular and renal vascular diseases and tumors; (2) Persons with mental illness or unable to communicate normally; (3) Complicated with acute and chronic infectious inflammation, fracture, tumor, secondary hypertension or other serious physical diseases.

### Index selection

The selected clinical data sources include patients’ general data, cardiac ultrasound recording, laboratory examination results. General patient information includes complications (hypertension, diabetes, cerebral infarction), bad living habits (smoking, drinking), demographic data (education level, payment method of medical expenses, monthly family income, marital status, age, gender, body mass index (BMI), systolic blood pressure at admission, diastolic blood pressure at admission, mean arterial pressure at admission, pulse pressure at admission). Cardiac ultrasound recording includes left ventricular ejection fraction (LVEF), left ventricular end-diastolic dimension (LVEDD). Laboratory examination indicators includes thyroid-stimulating hormone (TSH), triiodothyronine (FT3), free thyroxine (FT4), very low density lipoprotein cholesterol (VLDL-C), low density lipoprotein cholesterol (LDL-C), high density lipoprotein cholesterol (HDL-C), triglyceride, cholesterol, blood calcium, blood sodium, blood potassium, blood carbon dioxide binding capacity, uric acid (UA), blood urea nitrogen (BUN), albumin (ALB), aspartate amino transferase (AST), alanine aminotransferase (ALT), platelet count (PLT), hemoglobin (HGB), red blood cell count (RBC), white blood cell count (WBC), N-terminal pro-brain natriuretic peptide (NT-proBNP), C-reactive protein (CRP), D-Dimer, Fasting blood glucose at admission. The above data are collected from the electronic medical record system of the First Affiliated Hospital of University of science and technology of China.

### Statistical treatment

EpiData software version 3.1 (EpiData Association, Odense, Denmark) was used to create the database, the SPSS software program,version 24.0, for Windows (IBM Corp, Armonk, NY, USA) and R software (http://www.r-project.org; R Foundation for Statistical Computing, Vienna, Austria) were used to analyze the data. Univariate analysis was performed using the independent sample t-test, Mann Whitney rank sum test, uncorrected Pearson chi square test, and Fisher exact probability method. Logistic regression was used to examine the indicators that had statistical differences in univariate analysis. The “AMORE” package (Limas et al., 2020), “randomForest” package (Liaw & Wiener, 2002), and “xgboost” packages (Chen et al., 2021) in R software are used to create the BP neural network (BPNN) model, random forest (RF) model, and extreme gradient boosting (XGBoost) model, respectively. Different models were evaluated using prediction accuracy, sensitivity, specificity, F1 score, area under the receiver operating characteristic curve (AUC), positive predictive value, and negative predictive value. *P* < 0.05 indicated that the difference was statistically significant.

## Results

### Comparison of general data between the two groups

There are significant differences in the distribution of smoking, diabetes, payment method of medical expenses, monthly family income, gender, TSH, FT4, FT3, LDL-C, HDL-C, blood calcium, blood sodium, Bun, ALB, AST, ALT, WBC, NT-proBNP, CRP, D-Dimer, fasting blood glucose at admission, LVEDd, LVEF and age between the two groups (*P* < 0.05), as shown in Table 1.

**Table 1:** Comparison of relevant data between the two groups.

Variable	Total (*n* = 553)	Non-coronary heart disease group (*n* = 352)	Coronary heart disease group (*n* = 201)	Statistic	*P*
Smoking, *n* (%)				20.455^a^	<0.001
no	379 (68.54)	265 (75.28)	114 (56.72)		
yes	174 (31.46)	87 (24.72)	87 (43.28)		
Drinking wine/alcohol, *n* (%)				3.337^a^	0.068
no	397 (71.79)	262 (74.43)	135 (67.16)		
yes	156 (28.21)	90 (25.57)	66 (32.84)		
Hypertension, *n* (%)				0.214^a^	0.643
no	241 (43.58)	156 (44.32)	85 (42.29)		
yes	312 (56.42)	196 (55.68)	116 (57.71)		
Diabetes, *n* (%)				14.146^a^	<0.001
no	450 (81.37)	303 (86.08)	147 (73.13)		
yes	103 (18.63)	49 (13.92)	54 (26.87)		
Cerebral infarction, *n* (%)				1.860^a^	0.173
no	502 (90.78)	324 (92.05)	178 (88.56)		
yes	51 (9.22)	28 (7.95)	23 (11.44)		
Payment method of hospitalization expenses, *n* (%)				13.072^a^	0.004
At one’s own expense	75 (13.56)	56 (15.91)	19 (9.45)		
Employee medical insurance	179 (32.37)	99 (28.13)	80 (39.80)		
Resident medical insurance	273 (49.37)	184 (52.27)	89 (44.28)		
Provincial medical insurance	26 (4.70)	13 (3.69)	13 (6.47)		
Monthly household income, *n* (%)				43.877^a^	<0.001
3,000 yuan and below	367 (66.37)	269 (76.42)	98 (48.76)		
3,001–5,000 yuan	153 (27.67)	68 (19.32)	85 (42.29)		
More than 5,000 yuan	33 (5.97)	15 (4.26)	18 (8.96)		
Marriage, *n* (%)				–	0.437^b^
married	541 (97.83)	345 (98.01)	196 (97.51)		
divorce	5 (0.90)	4 (1.14)	1 (0.50)		
Widowed	6 (1.08)	3 (0.85)	3 (1.49)		
unmarried	1 (0.18)	0 (0.00)	1 (0.50)		
Education level, *n* (%)				2.630^a^	0.105
Junior high school and below	349 (63.11)	231 (65.63)	118 (58.71)		
High school and above	204 (36.89)	121 (34.38)	83 (41.29)		
Gender, *n* (%)				34.750^a^	<0.001
Male	355 (64.20)	194 (55.11)	161 (80.10)		
Female	198 (35.80)	158 (44.89)	40 (19.90)		
TSH (mlU/L)	2.51 (1.52, 3.93)	2.29 (1.47, 3.22)	3.60 (1.63, 4.62)	−4.765^c^	<0.001
FT4 (pmol/L)	11.74 (10.46, 13.11)	11.52 (10.30, 12.89)	12.04 (11.12, 13.49)	−3.268^c^	0.001
FT3 (pmol/L)	4.55 (3.95, 5.03)	4.73 (4.35, 5.16)	3.80 (1.58, 4.77)	9.355^c^	<0.001
VLDL-C (mmol/L)	0.92 (0.74, 1.15)	0.94 (0.74, 1.15)	0.90 (0.75, 1.18)	−0.281^c^	0.779
LDL-C (mmol/L)	2.34 (1.84, 2.91)	2.29 (1.83, 2.82)	2.39 (1.94, 3.16)	−2.167^c^	0.03
HDL-C (mmol/L)	1.02 (0.87, 1.20)	1.06 (0.92, 1.25)	0.94 (0.81, 1.09)	5.603^c^	<0.001
Triglyceride (mmol/L)	1.57 (1.11, 2.18)	1.51 (1.11, 2.12)	1.64 (1.14, 2.36)	−1.623^c^	0.105
Cholesterol (mmol/L)	4.35 (3.66, 5.09)	4.41 (3.68, 5.03)	4.27 (3.63, 5.23)	−0.376^c^	0.707
Blood calcium (mmol/L)	2.25 (2.16, 2.34)	2.26 (2.18, 2.33)	2.22 (2.09, 2.36)	2.810^c^	0.005
Blood sodium (mmol/L)	141.00 (139.00, 142.00)	141.00 (140.00, 142.00)	140.00 (138.00, 142.00)	6.094^c^	<0.001
Blood potassium (mmol/L)	3.90 (3.70, 4.14)	3.91 (3.71, 4.12)	3.89 (3.69, 4.18)	−0.369^c^	0.712
Blood carbon dioxide binding capacity (mmol/L)	25.00 (23.30, 27.00)	24.90 (23.20, 26.80)	25.30 (23.70, 27.20)	−1.739^c^	0.082
UA (mmol/L)	337.00 (265.00, 407.00)	337.00 (268.00, 405.00)	339.00 (263.00, 409.00)	−0.067^c^	0.947
BUN (mmol/L)	5.94 (4.98, 7.00)	6.10 (5.20, 7.02)	5.53 (4.58, 6.86)	3.244^c^	0.001
ALB (g/L)	43.10 (40.70, 45.30)	43.40 (41.40, 45.50)	42.20 (39.30, 45.00)	3.931^c^	<0.001
AST (U/L)	22.00 (17.00, 33.00)	20.00 (17.00, 25.00)	30.00 (20.00, 89.00)	−8.566^c^	<0.001
ALT (U/L)	23.00 (16.00, 40.00)	21.00 (14.00, 32.00)	33.00 (20.00, 50.00)	−7.290^c^	<0.001
PLT (10^9^/L)	199.00 (168.00, 240.00)	195.00 (166.00, 236.00)	204.00 (171.00, 246.00)	−1.113^c^	0.266
HGB (g/L)	135.00 (123.00, 146.00)	135.00 (124.00, 145.00)	135.00 (122.00, 147.00)	−0.136^c^	0.892
RBC (10^12^/L)	4.39±0.55	4.42±0.51	4.33±0.60	1.920^d^	0.056
WBC (10^9^/L)	6.55 (5.33, 8.13)	6.21 (5.18, 7.48)	7.70 (5.89, 9.64)	−6.192^c^	<0.001
NT-proBNP (pg/ml)	100.00 (50.00, 342.00)	50.00 (50.00, 106.00)	313.00 (100.00, 1209.00)	−12.737^c^	<0.001
CRP (mg/L)	5.00 (5.00, 7.80)	5.00 (5.00, 5.00)	5.00 (5.00, 10.00)	−3.933^c^	<0.001
D-Dimer (mg/L)	0.26 (0.20, 0.39)	0.25 (0.20, 0.33)	0.31 (0.21, 0.53)	−4.149^c^	<0.001
Fasting blood glucose at admission (mmol/L)	6.25 (5.39, 7.90)	6.03 (5.25, 7.02)	7.12 (5.83, 9.53)	−6.304^c^	<0.001
LVEDD (mm)	51.00 (49.00, 54.00)	50.00 (48.00, 53.00)	53.00 (50.00, 58.00)	−6.111^c^	<0.001
LVEF (%)	66.00 (60.00, 70.00)	67.00 (63.00, 71.00)	62.00 (52.00, 67.00)	7.864^c^	<0.001
BMI (kg/m^2^)	25.00 (23.00, 27.00)	25.00 (23.00, 28.00)	24.79 (23.24, 26.61)	0.365^c^	0.715
Mean arterial pressure at admission (mmHg)	98.00 (90.00, 107.00)	98.00 (90.00, 107.00)	97.33 (89.00, 107.00)	0.951^c^	0.342
Pulse pressure at admission (mmHg)	47.00 (37.00, 57.00)	47.00 (38.00, 56.00)	46.00 (36.00, 57.00)	0.256^c^	0.798
Diastolic blood pressure at admission (mmHg)	83.00 (75.00, 91.00)	83.00 (76.00, 91.00)	83.00 (74.00, 91.00)	0.979^c^	0.328
Systolic blood pressure at admission (mmHg)	129.00 (119.00, 143.00)	130.00 (120.00, 143.00)	127.00 (116.00, 144.00)	0.974^c^	0.33
Age (year)	55.00 (50.00, 60.00)	55.00 (49.00, 59.00)	56.00 (51.00, 62.00)	−2.306^c^	0.021

**Notes:**

a Uncorrected Pearson chi square test.

b Fisher exact probability method.

c Mann Whitney rank sum test.

d Independent sample t-test.

### Multivariate logistic regression analysis of the risk of coronary heart disease in middle-aged and young people

The incidence of coronary heart disease was used as the dependent variable, while 24 factors with *P* < 0.05 in Table 1 were used as independent variables in the multivariate logistic regression model, and the logistic regression method was used for variable screening by the backward method with the smallest Akaike information criterion (AIC). The results showed that age, blood glucose at admission, AST and LDL-C were independent risk factors for coronary heart disease in young and middle-aged people, and LVEF, ALB, Blood sodium, HDL-C and gender were independent protective factors for coronary heart disease in young and middle-aged people. As shown in Table 2.

**Table 2:** Multivariate Logistic regression analysis of the risk of coronary heart disease in middle-aged and young people.

Variables	Estimate	SE	Z	*P*	OR	Lower (95% CI)	Upper (95% CI)
Constant	21.030	8.839	2.379	0.017	–	–	–
Age	0.096	0.019	5.004	0.000	1.100	1.061	1.144
LVEF	−0.038	0.014	−2.699	0.007	0.963	0.936	0.989
Fasting blood glucose at admission	0.092	0.041	2.243	0.025	1.096	1.012	1.190
AST	0.013	0.003	3.733	0.000	1.013	1.007	1.020
ALB	−0.092	0.034	−2.712	0.007	0.912	0.852	0.974
Blood sodium	−0.128	0.059	−2.177	0.029	0.880	0.785	0.988
HDL-C	−2.145	0.597	−3.593	0.000	0.117	0.035	0.367
LDL-C	0.259	0.133	1.946	0.052	1.296	0.992	1.704
Gender (Reference = Male)	−0.864	0.325	−2.662	0.008	0.421	0.220	0.789

**Note:**

SE, Standard Error of regression coefficients of variables in regression model; Z, The Z statistic is used for hypothesis testing of OR values of variables in regression model; P, The probability values calculated by hypothesis testing of the OR values of the variables in the regression model; if *P* < 0.05, the OR value of the variable is considered statistically significant.; OR, Odds Ratio, A variable with an OR of 1 indicates that the variable has no significant effect on the outcome; a variable with an OR > 1 indicates that the variable has a large effect on the outcome (positive association); and a variable with an OR < 1 indicates that the variable is a protective factor, *i.e*., it is negatively associated with the outcome; CI, confidence interval of OR values of the variables in the regression model.

### Machine learning model

The 24 indicators with statistical differences between the two groups in Table 1 are included in three machine learning models. The test set (*n* = 82, 15.00%) are randomly selected from the overall sample, and the remaining samples are used as the training set for 10 fold cross validation, so as to train and verify the training set, and the test set was used to evaluate the classification ability of the samples. The performance evaluation indexes of different machine learning models in training set, validation set and test set are shown in Table 3. From the performance parameters of different machine learning models in the test set, the XGBoost model has the best performance, and the AUC and F1 scores of this model are higher than those of other algorithms.

**Table 3:** Performance evaluation index analysis of four models.

Evaluation indicator	Logistic regression model	BP neural network model	Stochastic forest model	XGBoost model
Training set				
AUC	0.863	0.777	1.000	1.000
Prediction accuracy	0.814	0.742	0.996	0.998
Sensitivity	0.740	0.780	1.000	1.000
Specificity	0.864	0.722	1.000	1.000
Positive predictive value	0.773	0.634	1.000	1.000
Negative predictive value	0.842	0.847	0.994	0.996
F1 score	0.753	0.693	1.000	1.000
Validation set				
AUC	0.841	0.792	0.969	0.983
Prediction accuracy	0.791	0.745	0.879	0.921
Sensitivity	0.839	0.849	0.972	0.972
Specificity	0.784	0.735	0.907	0.959
Positive predictive value	0.752	0.642	0.930	0.954
Negative predictive value	0.818	0.852	0.865	0.906
F1 score	0.789	0.728	0.949	0.962
Test set				
AUC	0.829	0.795	0.928	0.940
Prediction accuracy	0.747	0.687	0.880	0.867
Sensitivity	0.818	0.909	0.818	0.909
Specificity	0.705	0.639	0.869	0.836
Positive predictive value	0.517	0.455	0.875	0.867
Negative predictive value	0.870	0.949	0.881	0.868
F1 score	0.634	0.606	0.846	0.887

**Note:**

AUC, area under the receiver operating characteristic curve.

### Importance analysis of variables in different machine learning models

**Figure 1:**
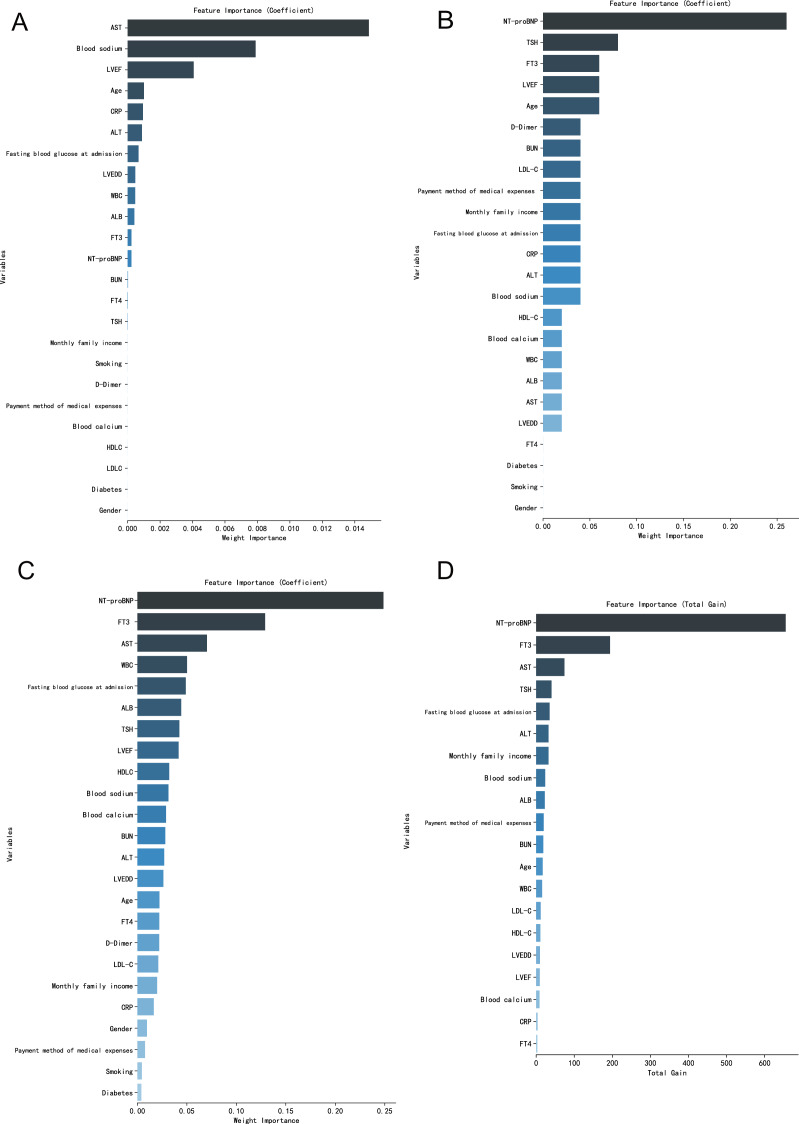
(A–D) Importance analysis of variables in different machine learning models.

From the order of relative importance of 24 indicators in logistic regression and three machine learning algorithms, the relative importance of the BP neural network model and random forest model indicators is relatively balanced, while the logistic regression model and XGBoost model are a few indicators with high relative importance (Fig. 1).

## Discussion

With the development and rupture of coronary atherosclerotic plaque in patients with coronary heart disease, it can lead to arterial thrombosis, acute myocardial infarction and life-threatening (Taha et al., 2018). With the change of diet structure, lifestyle and work rhythm of Chinese residents, patients with coronary heart disease tend to be younger, and the incidence rate of acute myocardial infarction among young and middle-aged patients has also significantly increased (Yanmei, 2019). There are certain differences in risk factors and coronary lesion characteristics in patients with coronary heart disease and acute myocardial infarction at different ages, which directly affect the effect of disease prevention and treatment (Li, Xi & Xiaotao, 2020). A review of the literature undertaken found that the proportion of overweight, smoking history, family history of coronary heart disease and drinking history in the middle-aged and young people is significantly higher than that in the elderly. The proportion of bad eating habits such as high salt and high fat in the middle-aged and young people is significantly higher than that in the elderly. The overweight rate also increases accordingly, which promotes the occurrence and development of coronary atherosclerosis (Chenghua, Shan & Jingbo, 2021). Therefore, we should strengthen the early screening and diagnosis of coronary heart disease in young and middle-aged people, and avoid many problems such as poor disease control caused by untimely detection and treatment.

Through traditional logistic regression analysis, it was found that age, fasting blood glucose at admission, AST and LDL-C were independent risk factors for coronary heart disease in middle-aged and young people, and LVEF, ALB, Blood sodium, HDL-C and gender were independent protective factors for coronary heart disease in middle-aged and young people. With the growth of age, the possible reason for the increased risk of coronary heart disease in young and middle-aged people is that the elderly patients have a long time of coronary artery disease, and the proportion of hypertension and hyperlipidemia is high, which is easy to cause the proliferation of subintimal smooth muscle, the dysfunction of myocardial energy metabolism, aggravate myocardial ischemia and hypoxia, and cause the occurrence of coronary artery disease (Chenghua, Shan & Jingbo, 2021). Studies have shown that the incidence of dangerous complications in patients with coronary heart disease in the early stage of diabetes (impaired glucose tolerance and impaired fasting glucose) is increased (Hu et al., 2002), and the degree of coronary artery disease is more serious with the increase of fasting glucose (Sevinc Ok et al., 2012). The study of domestic scholars found that fasting blood glucose in people without diabetes is related to the occurrence of coronary heart disease and the severity of coronary artery disease, and fasting blood glucose is a risk factor for coronary heart disease (Midiribuick et al., 2018), which is consistent with the results of this study. AST is a commonly used index to detect liver function. The increase of its level suggests that patients’ liver function is damaged to a certain extent, and serum AST can be used as an important index to judge the occurrence of coronary heart disease and the severity of other types of cardiovascular diseases (Yunlong & Yan, 2019). LDL-C is the most concerned blood lipid index in predicting atherosclerotic cardiovascular disease. The decrease of its value can benefit from the decrease of atherosclerotic cardiovascular endpoint (Yangjie, Kun & Xiufang, 2021; Schwartz et al., 2018). HDL-C is a common blood lipid index, which is mainly synthesized in the liver and has the effect of anti atherosclerosis. Its level is reduced, which can lead to abnormal lipid metabolism and accelerate the progress of coronary atherosclerosis (Lei, Xiaoyu & Zhongrui, 2019). LVEF is a common index to reflect the classification of cardiac function and left ventricular systolic function. Myocardial ischemia and hypoxia injury in CHD patients, cardiac overload work leads to the reduction of myocardial systolic function, LVEF and cardiac output (Hongmei, Guangli & Na, 2019). ALB is a non-specific transfer protein, which can combine with insoluble small molecules and inorganic ions to form a complex conducive to dissolution. Its level is reduced, which can cause abnormal transport of metabolic substances in patients, adhere to and precipitate in blood vessels, lead to the formation of vascular plaque and aggravate the degree of coronary artery stenosis (Bingrui, 2018). Some studies have shown that hyponatremia may also be a risk predictor for acute myocardial infarction (Bae et al., 2017; Burkhardt et al., 2015). Other studies have found that hyponatremia is quite common in patients with elevation myocardial infarction in the acute phase, which is related to many other baseline characteristics suggesting poor prognosis, especially serum Na^+^<130 mmol/L. The short-term mortality and the incidence of cardiogenic shock, heart failure and life-threatening arrhythmia in patients with serum Na^+^<130 mmol/L were significantly increased (Tao, Yanmin & Jun, 2017). Compared with men, the lower risk of coronary heart disease in young and middle-aged women may be due to the higher level of estrogen in young and middle-aged women, which can relax blood vessels, reduce low-density lipoprotein and fibrinogen, and reduce the risk of coronary heart disease (Haiqiu, Mei & Faxin, 2017).

Experts and scholars have begun some exploration on how to use machine learning algorithm to diagnose coronary heart disease. Data from the survey of chronic diseases in Jilin Province in China suggests that, three machine learning algorithms including support vector machine, random forest and neural network were selected and were used to establish the recognition model of coronary heart disease, with the optimal accuracy of 0.669 (Kai, 2016). It has been shown in the literature that the data of clinical symptoms, demographic information and living habits of patients in Shandong Province in China were collected, and a coronary heart disease screening model using support vector machine algorithm was established. The accuracy of the model is 0.894 (Chunyan, 2019). According to literature reports (Yi, 2018), the basic information, clinical symptoms and laboratory test data of subjects in Jinan qianfushan hospital were collected, and a coronary heart disease screening model by using heterogeneous ensemble learning method was established, with an accuracy of 0.963. A study on risk assessment models for coronary heart disease in the elderly showed that the risk assessment models for coronary heart disease in the elderly based on the medical examination data of the elderly in the community using logistic and XGBoost algorithms had good stability, among which the performance of the XGBoost algorithm model was better than that of the logistic algorithm model and could provide a methodological reference for the risk assessment of coronary heart disease in the elderly in the community (Xiaoli, Tianxing & Derong, 2021). However, there is no comprehensive study on the risk of coronary heart disease in specific middle-aged and young people from the perspective of machine learning in China.

By exploring the correlation between clinical indicators related to the occurrence of coronary heart disease and outcome events in young and middle-aged people, this study established the traditional logistic regression model and three other machine learning models. After comparison, it was finally found that the XGBoost model performed best and had a good discriminant effect on the occurrence of coronary heart disease in young and middle-aged people (AUC = 0.940, F1 score = 0.887). A research report on the risk of essential hypertension complicated with coronary heart disease, which is similar to the conclusion of this study, shows that the classification accuracy of the logistic regression classification model, random forest model and XGBoost model in the test set are 0.852, 0.966 and 0.976 respectively, and the AUC under receiver operating characteristic curve is 0.853, 0.967 and 0.977 respectively. The XGBoost model with the best performance was applied to the verification group, and the diagnostic accuracy was 0.926 and AUC was 0.956, which indicated that machine learning had a good application effect in predicting the risk of coronary heart disease and the XGBoost model established had a good auxiliary diagnostic function for essential hypertension complicated with coronary heart disease, and achieved good results in clinical practice (Jun, Chao & Xiaogang, 2020). The XGBoost algorithm is improved based on gradient descent tree algorithm. Compared with other machine learning algorithms, the XGBoost algorithm has the characteristics of fast training speed, high efficiency and strong generalization ability. It is widely used in the field of regression and classification (Huiping & Anmin, 2020). In the analysis of the relative importance of indicators, the XGBoost model has a high relative importance with a few indicators. Compared with the other two machine learning algorithms, the XGBoost model can use fewer indicators to achieve high accuracy. It is more practical in the case of incomplete or missing indicators in clinical practice. Therefore, through the performance evaluation of the model, it is considered that the individual risk prediction model of coronary heart disease in young and middle-aged people constructed by the XGBoost algorithm is the best.

## Conclusion and limitation

Compared with the other three machine learning algorithms, the XGBoost model is the best algorithm to predict the risk of coronary heart disease in young and middle-aged people, which is helpful for screening the high-risk population of coronary heart disease in young and middle-aged people according to early clinical characteristics. However, this study is only a single center study with limited sample size. In the future, it will be necessary to include a larger sample size for external validation test in order to further improve and improve the accuracy of the model.

## Supplemental Information

10.7717/peerj.14078/supp-1 Supplemental Information 1 Raw data.

## Additional Information and Declarations

### Data Availability

The following information was supplied regarding data availability: The raw data are available in the Supplemental Files.
